# Low-dose versus high-dose dexamethasone for hospitalized patients with COVID-19 pneumonia: A randomized clinical trial

**DOI:** 10.1371/journal.pone.0275217

**Published:** 2022-10-03

**Authors:** Huimin Wu, Salim Daouk, Jad Kebbe, Fawad Chaudry, Jarrod Harper, Brent Brown

**Affiliations:** 1 Pulmonary, Critical Care and Sleep Medicine Section, Department of Medicine, University of Oklahoma Health Sciences Center, Oklahoma City, Oklahoma, United States of America; 2 College of Medicine, University of Oklahoma Health Sciences Center, Oklahoma City, Oklahoma, United States of America; Srebrnjak Children’s Hospital, CROATIA

## Abstract

**Background:**

Dexamethasone 6 mg daily for 10 days is the recommended treatment for patients with severe or critical coronavirus disease 2019 (COVID-19). The evidence on the benefit of high-dose dexamethasone is limited. The goal of this study was to assess the effects of 6 mg daily vs. 20 mg daily of dexamethasone in hospitalized patients with COVID-19 pneumonia.

**Methods:**

We conducted a single-center, randomized, clinical trial involving hospitalized patients with COVID-19 pneumonia. Participants were randomized 1:1 to dexamethasone 6 mg daily or dexamethasone 20 mg daily, and were stratified by the WHO-Ordinal Scale for Clinical Improvement (OSCI). The primary outcome was clinical improvement equal to or greater than 2 points by OSCI on day 28. Secondary outcomes were 28-day mortality, intensive care unit-free days, and ventilator-free days on day 28.

**Results:**

Of the 107 patients who enrolled and completed the follow up, 55 patients enrolled in the low-dose group and 52 patients enrolled in the high-dose group. Treatment with dexamethasone 20 mg daily compared with dexamethasone 6 mg daily did not result in better clinical improvement based on OSCI on day 28 (71.2% vs. 78.2%; odds ratio, 1.45 [0.55–3.86]; p = 0.403). For participants who required high-flow oxygen or noninvasive ventilation at randomization, the 6-mg group had better survival than the 20-mg group on day 28 (100% vs. 57.1%; p = 0.025). Although more participants in the 6-mg group received immune modulators (40% vs. 21.2%; p = 0.035), 50% of death cases in the 20-mg group who required high-flow oxygen or noninvasive ventilation at randomization received immune modulators.

**Conclusions:**

Dexamethasone 20 mg daily did not result in better clinical outcome improvement, and was probably associated with higher 28-day mortality in patients on high-flow oxygen or noninvasive ventilation, compared with dexamethasone 6 mg daily.

**Trial registration:**

Clinialtrials.gov number, NCT04707534, registered January 13, 2021

## Background

Dexamethasone is the recommended treatment for patients with severe or critical coronavirus disease 2019 (COVID-19) [1]. Multiple clinical trials reported the benefit of administering systemic corticosteroids in hospitalized patients with COVID-19 [2]. In the RECOVERY trial, dexamethasone 6 mg once daily for up to 10 days reduced 28-day mortality in hospitalized patients with COVID-19 [3]. In the CoDEX trial, dexamethasone 20 mg daily for five days, followed by 10 mg daily for five days, increased the number of ventilator-free days [4]. While local practices vary, current guidelines recommend using dexamethasone 6 mg (intravenous or enterally) for ten days (or until discharge) for hospitalized patients with severe or critical COVID-19 [1].

Studies revealed that overwhelming inflammation is associated with severe and critical COVID-19 cases [5]. A higher dose of glucocorticoids has been used for non-COVID-19 acute respiratory distress syndrome (ARDS) [6]. A dose-response glucocorticoid effect on biomarkers of glucocorticoid receptor agonism has been observed in pharmacodynamic studies [7]. However, higher doses of glucocorticoids increase the risk of adverse reactions, particularly hyperglycemia and infections [6, 8]. This clinical trial was conducted to evaluate the efficacy and safety of a higher daily dose of dexamethasone in hospitalized patients with COVID-19 and hypoxemia. The hypothesis was that treatment with dexamethasone 20 mg daily is superior to dexamethasone 6 mg daily in achieving clinical improvement on day 28.

## Methods

### Study site and participant selection

This study was conducted at the University of Oklahoma Medical Center, Oklahoma, United States. Eligible participants were patients older than 18 years with PCR-confirmed COVID-19 infection on admission, needing supplemental oxygen administered via nasal cannula, face mask, high-flow nasal cannula, or positive pressure ventilation (noninvasive or invasive). Patients were excluded if they had been taking corticosteroids more than 48 hours prior to screening, had an underlying disease requiring chronic corticosteroids, had severe medical events before admission (e.g., cardiac arrest), had contraindication(s) for corticosteroids, or were recruited in other interventional clinical trials. Participation in registries was allowed. Patients were excluded if death was deemed imminent or inevitable during the subsequent 24 hours. Pregnant patients and patients on judicial protection were excluded.

The study was registered on clinialtrials.gov (ID: NCT04707534; registered January 13, 2021) and approved by the University of Oklahoma Health Sciences Center Institutional Review Board (IRB #:12927). Informed consent was obtained from participants or legally authorized representatives.

### Study design

The study was a pragmatic randomized parallel-group controlled open-label trial conducted between January 2021 and December 2021. Participants were randomly assigned in a 1:1 ratio to a high-dose dexamethasone group (20 mg daily for five days, followed by 10 mg daily for five days) or a low-dose dexamethasone group (6 mg once daily for ten days). Participants were randomized with a block size of 2 and 4 and stratified by the WHO-Ordinal Scale for Clinical Improvement (WHO-OSCI) [9]. The randomization sequence was generated using STATA 16 and uploaded into a secure web-based system (REDCap). REDCap assigned the treatment groups using the randomization sequence after the investigators entered the OSCIs in REDCap. The investigators did not know the randomization sequence until the treatment groups were assigned.

Subjects were screened and enrolled within 48 hours after the first dose of systemic corticosteroid. Randomization occurred shortly after the informed consent was signed.

The pragmatic nature of the trial allowed for primary providers and multidisciplinary team to change the dexamethasone dose and treatment duration if needed after randomization. Participation in the study ended after day 28 or at discharge from the hospital, whichever was earlier.

### Outcome assessment

The primary outcome was the clinical improvement at day 28 after randomization. Sustained clinical improvement was defined as an improvement equal to or greater than 2 points using the WHO-OSCI scale, as follows [9]: 1. Not hospitalized, no limitations on activities; 2. Not hospitalized, limitation on activities; 3. Hospitalized, not requiring supplemental oxygen; 4. Hospitalized, requiring supplemental oxygen by mask or nasal prongs; 5. Hospitalized, on non-invasive ventilation or high-flow oxygen devices; 6. Hospitalized, on invasive mechanical ventilation; 7. Hospitalized, on invasive mechanical ventilation + additional organ support (pressors, renal replacement therapy, extracorporeal membrane oxygenation); and 8. Death. Secondary outcomes were 28-day mortality, intensive care unit (ICU)-free days, and ventilator-free days on day 28.

### Data analysis

REDCap was used to store clinical data. Data were transferred from REDCap to STATA 16 to perform statistical analyses. Before analyses, data were screened for normality, outliers, and all other statistical test assumptions.

All analyses adhered to an intention-to-treat approach. Descriptive statistics were conducted to characterize the study population at baseline, the health outcomes, and adverse events. Between-group comparisons were made using the independent *t*-test or Mann-Whitney test (on non-normally distributed data) for two groups. Chi-squared tests and Fisher’s exact tests were used to evaluate the relationship between two categorical variables. Multivariable Cox regression was used to evaluate the overall survival. Prior studies indicated that COVID-19 clinical severity was associated with old age, male sex, and multiple chronic medical conditions [10–13]. Clinical trials also showed that tocilizumab and baricitinib improved survival [14, 15]. Age, gender, comorbidities (obesity, diabetes, lung disease, and heart disease), and immune modulator use were included in the Cox regression model. Kaplan-Meier survival curves and the log-rank test were used to describe the survival between groups. A two-tailed p-value <0.05 was considered statistically significant.

Clinical improvement was defined as an improvement equal to or greater than 2 points using the WHO-OSCI scale, and was examined on days 14 and 28. The actual days of hospital stay and WHO-OSCI were used for survival analysis.

The sample size was calculated based on evaluating change in the primary outcome, the WHO-OSCI, between the high- and low-dose groups. According to the RECOVERY trial [3], at least 67.2% of the patients had clinical improvement. Using a 2-sided α level of 0.05 and power of 80% to detect a difference of 15% improvement between groups, we estimated that 300 patients needed to be enrolled. The trial ended early because the interim subgroup data analysis found potential harm in the high-dose group (significantly higher mortality in the high-dose group with OSCI of 5 at randomization).

### Data and safety monitoring

Due to the urgent need for effective treatment, an internal data and safety monitoring board was appointed for monitoring the trial progress. The board included all project investigators. The board members met once per three months, usually by teleconference, to review data from this trial. The data board review included interim analysis of primary and secondary outcome data and dexamethasone adverse events. A special responsibility of the board was to review serious adverse events, as defined by deaths, life-threatening conditions, or events requiring permanent discontinuation of the treatment. If the high-dose dexamethasone group had a statistically significant worse health outcome compared with the control group, it might trigger an immediate suspension of the study. At the end of each board meeting, the board voted whether to continue the study as planned or whether to recommend changes to the study. The board had the option to recommend that the study be stopped early if there was evidence that the risk benefit ratio did not warrant continuation of the trial.

## Results

**Fig 1:**
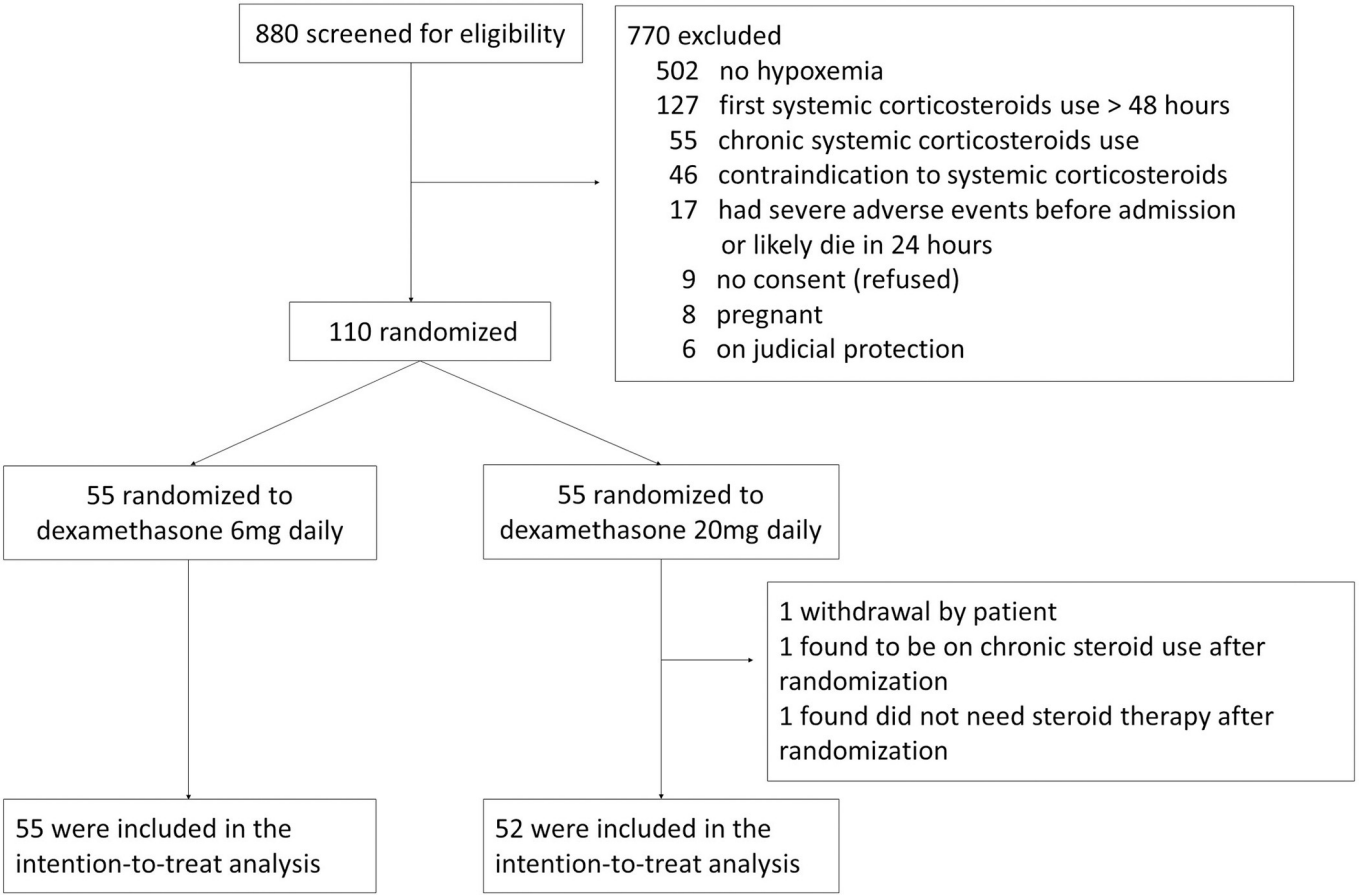
Consolidated standards of reporting trials diagram for flow of study participants.

A total of 110 patients were consented and enrolled in the trial. Three patients were excluded from the final data analysis (one patient withdrew after having consented; one patient was found to have been using chronic steroids after randomization; one patient was randomized and then found to not need steroid therapy). One hundred and seven patients were included for final data analysis (Fig 1). Baseline characteristics of participants in the low-dose and high-dose groups were similar, except there was a higher percentage of heart disease in the low-dose group (Table 1).

**Table 1:** Basic characteristics of study patients by dexamethasone dose group.

	Low-dose group (6 mg), n = 55	High-dose group (20 mg), n = 52
Age, yr mean (SD)	57.9 (16.9)	56.1 (14.2)
Male, n (%)	29 (52.7)	27 (51.9)
Race, n (%)		
White	24 (43.6)	29 (55.8)
Black or African American	17 (31.0)	15 (28.9)
Hispanic/Mexican American	10 (18.2)	5 (9.6)
American Indian or Alaska Native	2 (3.6)	2 (3.9)
Asian or Pacific Islander	1 (1.8)	1 (1.9)
Multiple Races/Other	1 (1.8)	0 (0)
BMI, mean (SD)	33.4 (9.7)	33.7 (9.3)
Past Medical History, n (%)		
Obesity	18 (32.7)	20 (38.5)
Diabetes	18 (32.7)	13 (25)
Chronic lung disease	9 (16.4)	8 (15.4)
Heart disease	20 (36.4)	10 (19.2)
Severe liver disease *	0 (0)	1 (1.9)
Severe kidney disease †	8 (14.6)	4 (7.7)
Current cancer	2 (3.6)	5 (9.6)
None of the above medical history	5 (9.1)	6 (11.5)
Days from first COVID symptoms to randomization, median (IQR)	7 (5–11)	8 (5–11)
Days from first COVID-19 test positive to randomization, median (IQR)	2 (1–6)	2 (1–6)
COVID vaccination history, n (%)‡		
Did not receive vaccination	48 (88.9)	46 (93.9)
Partially vaccinated	4 (7.40)	2 (4.08)
Fully vaccinated	2 (3.70)	1 (2.04)
OSCI at randomization, n (%)		
4	32 (58.2)	31 (59.6)
5	15 (27.3)	14 (26.9)
6	5 (9.1)	5 (9.6)
7	3 (5.5)	2 (3.9)
PaO_2_/FiO_2_ ratio, n (%)		
201–300	12 (21.8)	12 (23.1)
101–200	27 (49.1)	26 (50)
≤100	16 (29.1)	14 (26.9)
Serum ferritin, n (%)§		
High (>322 ng/ml)	41 (82.0)	34 (72.3)

IQR = interquartile range

PaO_2_/FiO_2_ = partial pressure of oxygen to fraction of inspired oxygen ratio

SD = standard deviation

* Severe liver disease was defined as requiring ongoing specialist care.

† Severe kidney impairment was defined as an estimated glomerular filtration rate of less than 30 ml per minute per 1.73 m^2^.

‡ 103 participants answered this question and were included in the analysis.

§ 97 participants had serum ferritin data at randomization.

The primary outcome was the clinical improvement of greater than or equal to 2 points using the WHO-OSCI (Table 2). There was no difference in WHO-OSCI change between the groups on days 14 and 28 after randomization. A similar proportion of participants reached clinical improvement in the low-dose group and the high-dose group on day 14 and day 28.

**Table 2:** Clinical improvement using the WHO-Ordinal scale for clinical improvement (WHO-OSCI).

	Low-dose group (6 mg), n = 55	High-dose group (20 mg), n = 52	Odds Ratio	95% Odds Ratio CI	P value
OSCI improvement on Day 14, median (IQR)* ‡	2 (0 to 3)	2 (-0.5 to 3)			0.231
OSCI improvement on Day 28, median (IQR)*§¶	0 (-1 to 3)	-0.15 (-3 to 3)			0.307
OSCI improvement ≥ 2 on Day 14, n (%)†‡	36 (65.5)	33 (63.5)	1.091	(0.458, 2.597)	0.830
OSCI improvement ≥ 2 on Day 28, n (%)†¶	43 (78.2)	37 (71.2)	1.453	(0.553, 3.862)	0.403

IQR = interquartile range

* use Mann-Whitney test

† Chi-square test

‡ if patients were discharged or died before day 14, the OSCI on discharge was used for the day 14 OSCI.

§ only includes patients who stayed in the hospital longer than 14 days after randomization.

¶If participants were discharged or died before Day 28, the OSCI on discharge or death was used for OSCI on Day 28.

**Fig 2:**
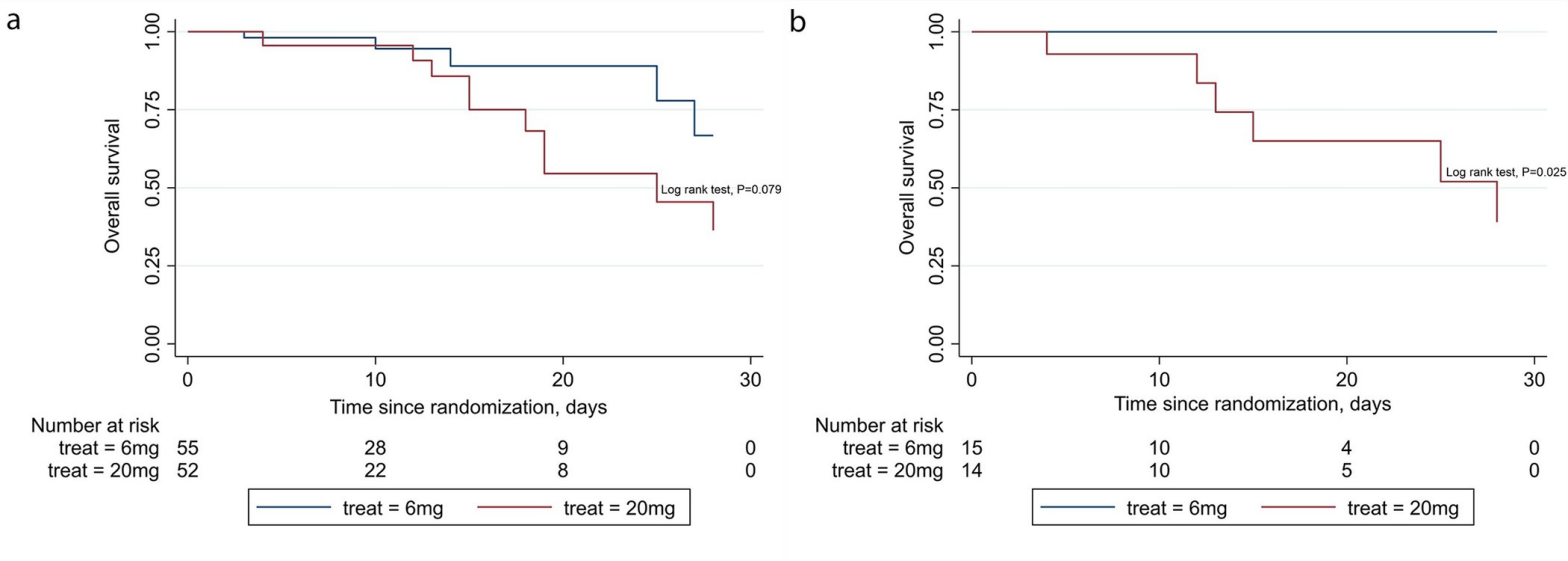
Kaplan-Meier survival curves a. in high-dose and low-dose groups and b. for subgroups with OSCI = 5 at randomization.

Mortality on day 28 was one of the secondary outcomes. Five participants (9.1%) in the low-dose group and 11 participants (21.2%) in the high-dose group died by day 28 (p = 0.080; Table 3, Fig 2A). In the subgroup analysis, for the participants who needed high-flow oxygen or noninvasive ventilation (OSCI = 5), all six deaths were from the high-dose group (Table 3, Fig 2B). Three patients died of severe ARDS and septic shock, two patients died of severe ARDS without septic shock, and one patient died after cardiac arrest possibly due to massive pulmonary embolism. There was no difference in mortality in other WHO-OSCI subgroups. Enrollment was halted after interim data analysis showed much higher mortality in the subgroup with a WHO-OSCI of 5 in the higher dose arm.

**Table 3:** Day 14 and day 28 death in WHO-Ordinal scale for clinical improvement (WHO-OSCI) subgroups.

	Participant number at randomization, n (%)		Death on Day 14, n		Death on Day 28, n	
OSCI*	Low-dose group (6 mg), n = 55	High-dose group (20 mg), n = 52	Low-dose group (6 mg), n = 55	High-dose group (20 mg), n = 52	Low-dose group (6 mg), n = 55	High-dose group (20 mg), n = 52
4	32 (58.2)	31 (59.6)	0	0	0	2
5	15 (27.3)	14 (26.9)	0	3	0	6
6	5 (9.1)	5 (9.6)	2	0	2	1
7	3 (5.5)	2 (3.9)	0	1	3	2

* OSCI at randomization

Multivariable Cox regression was conducted to evaluate the overall survival and dexamethasone use (S1 Table in the supporting information). Only younger age was associated with better overall survival after adjusting for gender, comorbidities, dexamethasone, and immune modulator use.

Thirty-eight participants needed ICU care in the first 28 days after randomization (Table 4). There was no difference in ICU-free days or ventilator-free days on day 28 between the low-dose group and high-dose group. The Sequential Organ Failure Assessment (SOFA) scores trended higher 48 hours after randomization in both groups. There was no difference in SOFA scores between groups at randomization or 48 hours later.

**Table 4:** Outcomes for participants who required ICU care within 28 days (n = 38).

		Low-dose group (6 mg), n = 19	High-dose group (20 mg), n = 19	P value
ICU-free days in first 28 days, median (IQR)*	0 (0–9)		0 (0–16)	0.256
Ventilator-free days in first 28 days, median (IQR)*	0 (0–23)		7 (0–18) †	0.516
SOFA Score, median (IQR)*				
At randomization		4 (2–8)	4 (2–5)	0.529
48 hours later		6 (2–11)	4 (3–8)	0.702

ICU = Intensive care unit

SOFA = Sequential Organ Failure Assessment

* use Mann-Whitney test

† two patients were not included due to Do Not Intubate status

For survivors, median hospital days were 9 days (interquartile range [IQR], 4–15) in the low dose-group and 6 days (IQR, 4–12) in the high-dose group (p = 0.538)

The incidence of adverse events, including hyperglycemia (requiring insulin infusion), pulmonary and systemic infections, and gastrointestinal bleeding, was low (S2 Table). Bacteremia was the most common adverse event with an incidence of 12%. There were no differences in adverse events between the groups.

Only five participants had changes in dexamethasone dose or duration when deemed in their best interest by their providers (S3 Table). Participants received additional SARS-CoV-2 therapies if they met relevant criteria (S4 Table). More participants in the low-dose group received immune moderator therapy (tocilizumab or baricitinib) than did participants in the high-dose group (40% vs. 21%; p = 0.035) (S4 and S5A Tables). Among the six deaths in the OSCI = 5 and high-dose dexamethasone subgroup, three patients received immune moderator therapy. The other three patients did not (S5B Table).

## Discussion

In this randomized clinical trial involving 107 adults with hypoxemia or acute respiratory failure associated with COVID-19, treatment with dexamethasone 20 mg daily for five days followed by 10 mg daily for five days compared with dexamethasone 6 mg daily for ten days did not result in better clinical improvement based on the WHO-OSCI. For participants who required high-flow oxygen or noninvasive ventilation, the 20-mg group had higher mortality than did the 6-mg group. There was no difference between groups in ICU-free days or ventilator-free days for patients requiring ICU care. The longer median hospital length of stay in the 6-mg group compared with the 20-mg group for survivors could be due to more patients surviving and needing longer hospitalization in the 6-mg group. The incidence of adverse events was low and similar in frequency in both groups. The high mortality in the high-dose and OSCI of 5 subgroup is a new finding compared with several previous studies and is an important warning about the “more is better” approach for corticosteroids use in our clinical practice.

Systemic corticosteroids are widely used for hospitalized patients with COVID-19 and hypoxemia. Several trials for assessing the therapeutic effects of different doses of systemic corticosteroids for COVID-19 showed various results. The COVID STEROID 2 trial evaluated the effect of dexamethasone 12 mg daily versus 6 mg daily in patients with COVID-19 and severe hypoxemia [16]. This trial did not reveal the benefit of days alive without life support at 28 days. In the secondary Bayesian analysis, they found high probabilities of benefit with dexamethasone 12 mg versus 6 mg on all outcomes, including the days alive without life support and mortality at days 28 and 90 [17]. However, this trial used a 12-mg daily dexamethasone dose in their high-dose arm, lower than our 20-mg daily dose. The 28-day mortality in this trial was higher (27.1% in the 12-mg group vs. 32.3% in the 6-mg group) than the mortality in our study. One explanation could be that our study enrolled younger patients; another could be that fewer participants required ICU care. Another open-label trial by Maskin et al. evaluated 16 mg daily of dexamethasone vs. 6 mg daily of dexamethasone in patients with COVID-19-related ARDS [18]. Although the trial was terminated due to low enrollment, there was no difference in ventilator-free days between groups with ARDS. Our study enrolled patients with various severities of acute hypoxemic respiratory failure and there was no difference in ventilator-free days in patients who needed ICU care, similar to the trial by Maskin et al. In addition, our trial found that patients who needed high flow oxygen or noninvasive mechanical ventilation and received high-dose dexamethasone had significantly higher mortality than did those in the low-dose dexamethasone group. It is a new finding and an important message for our clinical practice.

Taboada et al. compared dexamethasone 20 mg daily versus 6 mg daily and found that the groups who received the higher dose had reduced clinical worsening within 11 days after randomization [19]. An important difference from our trial was that this study only enrolled participants whose WHO-OSCI equaled 4. Patients with a WHO-OSCI of 5 or higher were not enrolled. Bouadma et al. also compared dexamethasone 20 mg daily versus 6 mg daily in patients who required oxygen support or invasive mechanical ventilation [20]. In this study, high-dose dexamethasone did not significantly improve 60-day survival.

Some clinical trials showed a benefit of higher dose of dexamethasone therapy [17, 19], while other trials reported opposite results. Toroghi et al. evaluated three different doses of dexamethasone (8 mg once daily, 8 mg twice daily, or 8 mg three times daily) for patients with moderate to severe COVID-19 [21]. Although stratification by severity was not used for the randomization, this study found the chance for a clinical response and the survival were significantly higher in the 8 mg once daily group than in the 8 mg three times daily group.

A systematic review and meta-analysis of high-dose versus low-dose corticosteroids in patients with COVID-19, including 12 observational studies and randomized controlled trials, demonstrated that high-dose corticosteroids did not reduce mortality [22]. The authors also admitted the limitation of a high degree of heterogeneity due to inconsistency of the dosage, types, and regimens among the reported studies and lack of subgroup analysis based on severity due to limited data.

In our study’s subgroup of individuals with a WHO-OSCI of 5, all six cases of death received dexamethasone 20 mg daily. There was no difference between death cases and survivors in terms of age, gender, medical comorbidities, or use of remdesivir or immune modulators. There was no difference in the incidence of corticosteroid adverse events. We suspect that the higher dose of corticosteroids may have over suppressed the normal antiviral responses in patients needing high-flow oxygen or noninvasive ventilation on admission. Caution must be taken when prescribing high dose systemic corticosteroids for this subgroup of patients in clinical practice.

The strengths of this trial include that participants with differing severities of acute hypoxemia were enrolled and stratified by OSCI at randomization. The pragmatic protocol ensured that the multidisciplinary team had flexibility in participant management, enhancing enrollment and completion of the study protocol. Our trial’s limitations include the single-center location, follow-up limited to while participants were in the hospital until day 28, and lack of knowledge of long-term health outcomes. This trial stopped early because the interim data analysis revealed that one subgroup (OSCI 5) in the high-dose arm had significantly higher mortality, which led to the small sample size. We were not able to draw conclusions for other OSCI subgroups. There is a small chance that the higher mortality may be a false-positive due to the small sample size. However, it would not have been ethical to continue enrolling more participants. There were more participants with heart disease in the low-dose group. More participants in the low-dose group received immune modulator therapy. These imbalances may affect the health outcomes. Although the multivariable regression to estimate the survival did not show a statistically significant difference after adjustment for heart disease and immune modulator use, it is well known that immune modulator therapy may improve survival. Half of the deaths in the high-dose group with OSCI = 5 at randomization received immune modulators. The other half did not. It was the same for those surviving. It is unlikely that not receiving immune modulators is the cause of the high mortality in this particular subgroup.

## Conclusions

Dexamethasone 20 mg once daily compared with dexamethasone 6 mg once daily did not result in significantly better clinical improvement in hospitalized patients with COVID-19-related acute hypoxemia at Day 28 after randomization. Dexamethasone 20 mg daily was probably associated with higher 28-day mortality in patients who needed high-flow oxygen or noninvasive ventilation. Caution must be taken when we prescribe high dose corticosteroids to this subgroup of patients.

## Supporting information

S1 Checklist CONSORT checklist. (PDF)

S1 Table Multivariable Cox regression analysis of overall survival. (DOCX)

S2 Table Hyperglycemia and infection events in the low-dose group and high-dose group. (DOCX)

S3 Table Dexamethasone treatment change in the hospital stay. (DOCX)

S4 Table Other COVID treatments. (DOCX)

S5 Table Participants who received immune modulators in each subgroup. (DOCX)

S1 File Trial study protocol (approved by ethics committee). (PDF)

S1 Dataset Minimal data set. (DTA)

## References

[1] Infectious Diseases Society of America. IDSA Guidelines on the Treatment and Management of Patients with COVID-19. Updated May 10, 2022. Accessed May 17, 2022, https://www.idsociety.org/practice-guideline/covid-19-guideline-treatment-and-management/

[2] WHO Rapid Evidence Appraisal for COVID-19 Therapies (REACT) Working Group. Association Between Administration of Systemic Corticosteroids and Mortality Among Critically Ill Patients With COVID-19: A Meta-analysis. *JAMA*. 2020;324(13):1330–1341. doi: 10.1001/jama.2020.17023 32876694 PMC7489434

[3] Horby P, Lim WS, Emberson JR, Mafham M, Bell JL, Linsell L, et al. Dexamethasone in Hospitalized Patients with Covid-19. *N Engl J Med*. Feb 25 2021;384(8):693–704. doi: 10.1056/NEJMoa2021436 32678530 PMC7383595

[4] Tomazini BM, Maia IS, Cavalcanti AB, Berwanger O, Rosa RG, Veiga VC, et al. Effect of Dexamethasone on Days Alive and Ventilator-Free in Patients With Moderate or Severe Acute Respiratory Distress Syndrome and COVID-19: The CoDEX Randomized Clinical Trial. *Jama*. Oct 6 2020;324(13):1307–1316. doi: 10.1001/jama.2020.17021 32876695 PMC7489411

[5] Lucas C, Wong P, Klein J, Castro TBR, Silva J, Sundaram M, et al. Longitudinal analyses reveal immunological misfiring in severe COVID-19. *Nature*. Aug 2020;584(7821):463–469. doi: 10.1038/s41586-020-2588-y 32717743 PMC7477538

[6] Steinberg KP, Hudson LD, Goodman RB, Hough CL, Lanken PN, Hyzy R, et al. Efficacy and safety of corticosteroids for persistent acute respiratory distress syndrome. *N Engl J Med*. Apr 20 2006;354(16):1671–84. doi: 10.1056/NEJMoa051693 16625008

[7] Fleishaker DL, Mukherjee A, Whaley FS, Daniel S, Zeiher BG. Safety and pharmacodynamic dose response of short-term prednisone in healthy adult subjects: a dose ranging, randomized, placebo-controlled, crossover study. *BMC Musculoskeletal Disorders*. 2016/07/16 2016;17(1):293. doi: 10.1186/s12891-016-1135-3 27424036 PMC4947329

[8] Raut A, Huy NT. Rising incidence of mucormycosis in patients with COVID-19: another challenge for India amidst the second wave? *Lancet Respir Med*. Aug 2021;9(8):e77. doi: 10.1016/S2213-2600(21)00265-4 34090607 PMC8175046

[9] World Health Organization. WHO R&D Blueprint. Novel Coronavirus: COVID-19 Therapeutic Trial Synopsis. Accessed Feb 18, 2022. https://www.who.int/blueprint/priority-diseases/key-action/COVID-19_Treatment_Trial_Design_Master_Protocol_synopsis_Final_18022020.pdf

[10] Bennett TD, Moffitt RA, Hajagos JG, Amor B, Anand A, Bissell MM, et al. Clinical Characterization and Prediction of Clinical Severity of SARS-CoV-2 Infection Among US Adults Using Data From the US National COVID Cohort Collaborative. *JAMA Netw Open*. 2021;4(7):e2116901. doi: 10.1001/jamanetworkopen.2021.16901 34255046 PMC8278272

[11] Aveyard P, Gao M, Lindson N, Hartmann-Boyce J, Watkinson P, Young D, et al. Association between pre-existing respiratory disease and its treatment, and severe COVID-19: a population cohort study. *Lancet Respir Med*. 2021;9(8):909–23. doi: 10.1016/S2213-2600(21)00095-3 33812494 PMC8016404

[12] Ioannou GN, Locke E, Green P, Berry K, O’Hare AM, Shah JA, et al. Risk Factors for Hospitalization, Mechanical Ventilation, or Death Among 10 131 US Veterans With SARS-CoV-2 Infection. *JAMA Netw Open*. 2020;3(9):e2022310. doi: 10.1001/jamanetworkopen.2020.22310 32965502 PMC7512055

[13] Freaney PM, Shah SJ, Khan SS. COVID-19 and Heart Failure With Preserved Ejection Fraction. *Jama*. 2020;324(15):1499–500. doi: 10.1001/jama.2020.17445 33001179

[14] RECOVERY Collaborative Group. Tocilizumab in patients admitted to hospital with COVID-19 (RECOVERY): a randomised, controlled, open-label, platform trial. *Lancet*. 2021;397(10285):1637–45. doi: 10.1016/S0140-6736(21)00676-0 33933206 PMC8084355

[15] Marconi VC, Ramanan AV, de Bono S, Kartman CE, Krishnan V, Liao R, et al. Efficacy and safety of baricitinib for the treatment of hospitalised adults with COVID-19 (COV-BARRIER): a randomised, double-blind, parallel-group, placebo-controlled phase 3 trial. *Lancet Respir Med*. 2021;9(12):1407–18. doi: 10.1016/S2213-2600(21)00331-3 34480861 PMC8409066

[16] Munch MW, Myatra SN, Vijayaraghavan BKT, Saseedharan S, Benfield T, Wahlin RR, et al. Effect of 12 mg vs 6 mg of Dexamethasone on the Number of Days Alive Without Life Support in Adults With COVID-19 and Severe Hypoxemia: The COVID STEROID 2 Randomized Trial. *Jama*. Nov 9 2021;326(18):1807–1817. doi: 10.1001/jama.2021.18295 34673895 PMC8532039

[17] Granholm A, Munch MW, Myatra SN, Vijayaraghavan BKT, Cronhjort M, Wahlin RR, et al. Dexamethasone 12 mg versus 6 mg for patients with COVID-19 and severe hypoxaemia: a pre-planned, secondary Bayesian analysis of the COVID STEROID 2 trial. *Intensive Care Med*. 2022;48(1):45–55. doi: 10.1007/s00134-021-06573-1 34757439 PMC8579417

[18] Maskin LP, Bonelli I, Olarte GL, Palizas F Jr., Velo AE, Lurbet MF, et al. High- Versus Low-Dose Dexamethasone for the Treatment of COVID-19-Related Acute Respiratory Distress Syndrome: A Multicenter, Randomized Open-Label Clinical Trial. *J Intensive Care Med*. Dec 13 2021:8850666211066799. doi: 10.1177/08850666211066799 34898320 PMC8926919

[19] Taboada M, Rodríguez N, Varela PM, Rodríguez MT, Abelleira R, González A, et al. Effect of high versus low dose of dexamethasone on clinical worsening in patients hospitalised with moderate or severe COVID-19 Pneumonia: an open-label, randomised clinical trial. *Eur Respir J*. Dec 16 2021; doi: 10.1183/13993003.02518–2021 PMC8678498 34916266

[20] Bouadma L, Mekontso-Dessap A, Burdet C, Merdji H, Poissy J, Dupuis C, et al. High-Dose Dexamethasone and Oxygen Support Strategies in Intensive Care Unit Patients With Severe COVID-19 Acute Hypoxemic Respiratory Failure: The COVIDICUS Randomized Clinical Trial. *JAMA Intern Med*. 2022 Jul 5. doi: 10.1001/jamainternmed.2022.2168 35788622 PMC9449796

[21] Toroghi N, Abbasian L, Nourian A, Davoudi-Monfared E, Khalili H, Hasannezhad M, et al. Comparing efficacy and safety of different doses of dexamethasone in the treatment of COVID-19: a three-arm randomized clinical trial. *Pharmacol Rep*. 2022 Feb;74(1):229–40. doi: 10.1007/s43440-021-00341-0 34837648 PMC8627167

[22] Tan RSJ, Ng KT, Xin CE, Atan R, Yunos NM, Hasan MS. High-Dose versus Low-Dose Corticosteroids in COVID-19 Patients: a Systematic Review and Meta-analysis. *J Cardiothorac Vasc Anesth*. 2022. doi: 10.1053/j.jvca.2022.05.011 35715291 PMC9101704

