# Supplementary material for: Low-dose versus high-dose dexamethasone for hospitalized patients with COVID-19 pneumonia: A randomized clinical trial
Source: PLoS One. 2022 Oct 3;17(10):e0275217. doi: 10.1371/journal.pone.0275217 (PMC9529091; doi:10.1371/journal.pone.0275217)
Supplement: S1 Table — (DOCX) [file pone.0275217.s002.docx]

**Supporting Information**

**S1 Table. Multivariable Cox regression analysis of overall survival**

| Variable | Hazard ratio (95% CI) | P value |
| --- | --- | --- |
| Age | 0.99 (0.98, 0.99) | 0.021 |
| Sex |  |  |
| Female | 1.00 | - |
| Male | 0.90 ((0.30, 2.69) | 0.848 |
| Obesity |  |  |
| No | 1.00 | - |
| Yes | 0.68 (0.18, 2.61) | 0.576 |
| Diabetes mellitus |  |  |
| No | 1.00 | - |
| Yes | 0.66 (0.20, 2.16) | 0.493 |
| Chronic lung disease |  |  |
| No | 1.00 | - |
| Yes | 1.05 (0.21, 5.19) | 0.949 |
| Heart disease |  |  |
| No | 1.00 |  |
| Yes | 2.35 (0.56, 9.84) | 0.242 |
| Dexamethasone 20mg |  |  |
| No | 1.00 | - |
| Yes | 2.35 (0.68, 8.02) | 0.174 |
| Immune modulator |  |  |
| No | 1.00 | - |
| Yes | 0.91 (0.29, 2.92) | 0.866 |
