# Supplementary material for: Low-dose versus high-dose dexamethasone for hospitalized patients with COVID-19 pneumonia: A randomized clinical trial
Source: PLoS One. 2022 Oct 3;17(10):e0275217. doi: 10.1371/journal.pone.0275217 (PMC9529091; doi:10.1371/journal.pone.0275217)
Supplement: S2 Table — (DOCX) [file pone.0275217.s003.docx]

**Supporting Information**

**S2 Table. Hyperglycemia and infection events in the low-dose group and high-dose group**

|  | Low-dose group (6 mg), n= 55 | High-dose group (20 mg), n=52 |
| --- | --- | --- |
| Hyperglycemia, requires insulin infusion | 1 (1.8) | 2 (3.9) |
| Bacteremia | 10 (18.2) | 3 (5.8) |
| Ventilator associated pneumonia | 2 (3.6) | 1 (1.9) |
| Catheter-related bloodstream infection | 3 (5.5) | 1 (1.9) |
| Candidemia | 1 (1.8) | 0 (0) |
| Pulmonary aspergillosis | 1 (1.8) | 0 (0) |
| Gastrointestinal bleeding | 1 (1.8) | 0 (0) |
