# Supplementary material for: Low-dose versus high-dose dexamethasone for hospitalized patients with COVID-19 pneumonia: A randomized clinical trial
Source: PLoS One. 2022 Oct 3;17(10):e0275217. doi: 10.1371/journal.pone.0275217 (PMC9529091; doi:10.1371/journal.pone.0275217)
Supplement: S3 Table — (DOCX) [file pone.0275217.s004.docx]

**Supporting Information**

**S3 Table. Dexamethasone treatment change in the hospital stay ^*^**

|  | Low-dose group (6 mg), n= 55 | High-dose group (20 mg), n=52 |
| --- | --- | --- |
| No change in the intervention | 52 | 48 |
| Early stop | 2 | 2 |
| Reason | Gastrointestinal bleeding (1)  Death (1) | Withdrawal of care (1)  Death (1) |
| Dose change | 1 | 2 |
| Reason | Dose was increased by primary treating physician (1) | Dose was reduced due to hyperglycemia (2) |

^*^ The dose and duration may change on the discharge by the primary treating physicians.
