# Supplementary material for: Low-dose versus high-dose dexamethasone for hospitalized patients with COVID-19 pneumonia: A randomized clinical trial
Source: PLoS One. 2022 Oct 3;17(10):e0275217. doi: 10.1371/journal.pone.0275217 (PMC9529091; doi:10.1371/journal.pone.0275217)
Supplement: S4 Table — (DOCX) [file pone.0275217.s005.docx]

**Supporting Information**

**S4 Table. Other COVID treatments**

|  | Low-dose group (6 mg), n= 55 | High-dose group (20 mg), n=52 |
| --- | --- | --- |
| Remdesivir | 38 (69.1) | 41 (78.9) |
| Immune modulator * | 22 (40.0) | 11 (21.2) |
| Monoclonal antibody | 2 (3.6) | 1 (1.9) |
| Convalescent plasma | 0 (0) | 1 (1.9) |
| Did not receive any COVID-related treatment except for dexamethasone | 11 (20.0) | 10 (19.2) |

* include tocilizumab and baricitinib
