# Supplementary material for: Low-dose versus high-dose dexamethasone for hospitalized patients with COVID-19 pneumonia: A randomized clinical trial
Source: PLoS One. 2022 Oct 3;17(10):e0275217. doi: 10.1371/journal.pone.0275217 (PMC9529091; doi:10.1371/journal.pone.0275217)
Supplement: S5 Table — (DOCX) [file pone.0275217.s006.docx]

**Supporting Information**

**S5 Table. Participants who received immune modulators in each subgroup**

1. **All participants**

| OSCI | Low-dose group (6 mg), n= 55 | | High-dose group (20 mg), n=52 | |
| --- | --- | --- | --- | --- |
|  | Received immune modulators | Did not receive immune modulators | Received immune modulators | Did not receive immune modulators |
| 4 | 5 | 27 | 3 | 28 |
| 5 | 12 | 3 | 7 | 7 |
| 6 | 3 | 2 | 1 | 4 |
| 7 | 2 | 1 | 0 | 2 |
| Total | 22 | 33 | 11 | 41 |

1. **Participants who had OSCI =5 on randomization and died on day 28**

| Low-dose group (6 mg), n= 0 | | High-dose group (20 mg), n=6 | |
| --- | --- | --- | --- |
| Received immune modulators | Did not receive immune modulators | Received immune modulators | Did not receive immune modulators |
| 0 | 0 | 3 | 3 |

OSCI = Ordinal Scale for Clinical Improvement
