# Supplementary material for: Low-dose versus high-dose dexamethasone for hospitalized patients with COVID-19 pneumonia: A randomized clinical trial
Source: PLoS One. 2022 Oct 3;17(10):e0275217. doi: 10.1371/journal.pone.0275217 (PMC9529091; doi:10.1371/journal.pone.0275217)
Supplement: S1 File — (PDF) [file pone.0275217.s007.pdf]

Title of Project: Effect of higher vs. lower doses of dexamethasone for COVID-19

Principal Investigator: Huimin Wu, MD MPH, Dept of Medicine
Co-Investigators: Brent Brown, MD; Fawad Chaudry; Salim Daouk, MD; Jad Kebbe, MD

Abstract
The coronavirus disease 2019 (COVID-19) pandemic is a serious global health threat. Multiple antiviral or immunomodulatory therapies have failed to show any mortality benefit for patients with COVID-19. Dexamethasone was shown in a large clinical trial to improve mortality. However, it is unclear what dose of dexamethasone is most beneficial in hospitalized patients with COVID-19. We plan to conduct a randomized single center open label clinical trial to evaluate two different doses of dexamethasone (20mg vs. 6mg) on the health outcome for hospitalized patients with COVID-19. The intervention arm is dexamethasone 20mg daily for 5 days, followed by dexamethasone 10mg daily for 5 days. The comparator is dexamethasone 6mg daily for 10 days. Three hundred participants will be enrolled. The primary outcome is clinical improvement using World Health Organization ordinal scale at day 28 or live discharge, whichever comes first. Our hypothesis is that patients who are treated with dexamethasone 20mg daily are more likely to have clinical improvement compare to those who are treated with dexamethasone 6mg daily on day 28.

A. Specific Aims

Studies showed dexamethasone has the mortality benefit and increases in the number of ventilator-free days for patients with COVID-19. However, there is no published trial to compare the health outcome for COVID-19 patients who are treated with different doses of dexamethasone. The primary aim of this study is to test the effectiveness of two difference dexamethasone dose to treat COVID-19. The hypothesis is that dexamethasone 20mg daily treatment is superior over dexamethasone 6mg daily in clinical improvement at day 28 using WHO ordinal scale for clinical improvement (1, 2)

B. Background and Significance

As of December 2020, COVID-19 - caused by severe acute respiratory syndrome coronavirus 2 (SARS-CoV-2) - has infected more than 20 million people with more than 350,000 deaths in the United States. (3) Several pharmacologic therapies for use in patients with COVID-19 have been investigated in randomized trials. Most antiviral or immunomodulatory therapies investigated have failed to show any mortality benefit. Only dexamethasone showed to improve mortality in RECOVERY trial. (4) In this randomized, controlled, open-label trial, dexamethasone 6 mg once daily (intravenous or by mouth) for up to 10 days reduced 28-day mortality in hospitalized patients with COVID-19. Another multicenter trial (CoDEX) using dexamethasone 20mg daily for 5 days, followed

Human Research Participant Protection The University of Oklahoma IRB APPROVED AAHRPP
IRB NUMBER: 12927
IRB APPROVAL DATE: 01/15/2021

by 10mg daily for 5 days revealed dexamethasone increase in the number of ventilator-free days.(5) However, there is no published trial to evaluate the difference of health outcomes using different dose of dexamethasone. Currently OUHSC COVID-19 Task Force recommends dexamethasone 6mg daily for 10 days for all hospitalized COVID-19 patients regardless of the respiratory status. (6) However, the COVID-19 morbidity and mortality remain high despite the recommended therapy. We plan to conduct a single center open label randomized clinical trial to investigate the heath outcomes in hospitalized patients with COVID-19 treated with dexamethasone 20mg daily for 5 days, 10mg daily for 5 days (high dose) vs. dexamethasone 6mg daily for 10 days (low dose).

C. Preliminary Studies/Progress Report

In RECOVERY trial, 2104 patients were assigned to receive dexamethasone 6mg daily for 10 days and 4321 to receive usual care (no dexamethasone). (4) Overall, 482 patients (22.9%) in the dexamethasone group and 1110 patients (25.7%) in the usual care group died within 28 days after randomization (age-adjusted rate ratio, 0.83; 95% confidence interval [CI], 0.75 to 0.93; P<0.001). In the dexamethasone group, the incidence of death was lower than that in the usual care group among patients receiving invasive mechanical ventilation (29.3% vs. 41.4%; rate ratio, 0.64; 95% CI, 0.51 to 0.81) and among those receiving oxygen without invasive mechanical ventilation (23.3% vs. 26.2%; rate ratio, 0.82; 95% CI, 0.72 to 0.94) but not among those who were receiving no respiratory support at randomization (17.8% vs. 14.0%; rate ratio, 1.19; 95% CI, 0.91 to 1.55). This trial concluded that in patients hospitalized with Covid-19, the use of dexamethasone resulted in lower 28-day mortality among those who were receiving either invasive mechanical ventilation or oxygen alone at randomization but not among those receiving no respiratory support.

In CoDex trial, 151 patients received 20mg of dexamethasone daily for 5 days, 10mg of dexamethasone daily for 5 days or until ICU discharge and standard care, 148 patients received standard care only.(5) Patients in the dexamethasone group had a mean 6.6 ventilator-free days (95% CI, 5.0-8.2) during the first 28 days vs 4.0 ventilator-free days (95% CI, 2.9-5.4) in the standard care group (difference, 2.26; 95% CI, 0.2-4.38; P = 0.04). Thirty-three patients (21.9%) in the dexamethasone group vs 43 (29.1%) in the standard care group experienced secondary infections, 47 (31.1%) vs 42 (28.3%) needed insulin for glucose control, and 5 (3.3%) vs 9 (6.1%) experienced other serious adverse events. It was concluded that among patients with COVID-19 and moderate or severe ARDS, use of intravenous dexamethasone plus standard care compared with standard care alone resulted in a statistically significant increase in the number of ventilator-free days (days alive and free of mechanical ventilation) over 28 days.

D. Research Design and Methods (What, When, How, Where)

1. Identify sources of research material in the form of biospecimens, records and/or data from interaction with participants.
   The electronic medical records demographic and clinical data on participants

Human Research Participant Protection The University of Oklahoma IRB APPROVED AAHRPP
IRB NUMBER: 12927
IRB APPROVAL DATE: 01/15/2021

will be collected and analyzed.

2. Describe study design, including sequence and timing of study procedures. Distinguish research procedures from those that are routine care. Provide a flow diagram or timetable.
    - It is a single center open label randomized clinical trial to investigate the different dose of dexamethasone on the health outcomes in hospitalized moderate or severe COVID patients.
    - It is a superiority trial.
    - This trial has two parallel groups. The participants will be randomly assigned in a 1:1 ratio to intervention group and control group.
    - We anticipate that approximately 300 participants will be enrolled and complete the study (Each group will enroll 150 participants.) The study plan to start as soon as the OUHSC IRB approves it and the duration is 28 days.
    - The COVID patients will be screened in the first 24 hours after investigators are contacted.
    - Participants will be randomized to intervention arm or control arm after participants agree to be enrolled into the study and sign the informed consent.
    - Except for the different doses of dexamethasone, participants will receive the routine medical care in OUMC.

Eligible individuals → Randomize
- → Dexamethasone 20mg + Standard care → Outcome
- → Dexamethasone 6mg + Standard care → Outcome

3. Include the study duration and number of study visits required.
This study will last for 5 months. There is only one visit for patients who are in OUMC for more than 28 days after randomization. There are two visits for patients who are not in OUMC at day 28 after randomization, first visit is the same as prior mentioned first visit, the second visit is a phone visit. The first visit is for screening, informed consent. After the visit, participants will be randomized and receive dexamethasone 20mg daily vs. 6mg daily. Participants will receive other routine medical care in the hospital till discharge. Research staff will review the hospital medical records daily to monitor the clinical improvement and any adverse event. At day 28, if participants are not in OUMC, research team will contact participants to evaluate the WHO ordinal scale.
4. Describe blinding, including justification for blinding or not blinding the trial.
This is an open label trial. The study outcome is objective. Not blinding the trial does not affect the study outcome.
5. Include justification for inclusion of a placebo or non-treatment group.
This study does not include a placebo or non-treatment group. At least two

Human Research Participant Protection
The University of Oklahoma
IRB APPROVED
AAHRPP
IRB NUMBER: 12927
IRB APPROVAL DATE: 01/15/2021

large clinical trials showed corticosteroids have benefit to COVID patients. Using placebo or non-treatment is not ethic.

6. Describe what happens to participants receiving therapy when study ends or if their participation in the study ends prematurely.
   Participation of the study ends after day 28. The investigator will not contact the participants or give other interventions or suggestions after day 28. Participants are welcome to contact the PI for any questions after study participation ends.
7. Indicate where the procedures will be completed.
   a. Include all sites where research activities will be conducted (consenting, interventions, chart reviews, data analysis, etc).
      This research will be conducted in University of Oklahoma Health Sciences Center. Consenting and interventions will be conducted in University of Oklahoma Medical Center. Chart reviews and data analysis will be conducted in University of Oklahoma Medical Center and College of Medicine.
   b. Indicate that all required approvals are already obtained or will be obtained at each research location prior to project implementation.
      This trial will be conducted at OUMC adult service. OUHSC IRB approval is needed. No other approval is needed. We will collaborate with other clinical services including emergency department and hospitalist service for participant recruitment. Dr. Brent Brown, study Co-PI and chief of pulmonary and critical care section will facilitate the collaborations.
8. Indicate whether clinically relevant research results, including individual research results, will be disclosed to participants. If so, under what conditions?
   The individual research results, including the oxygen support information, the length of hospital stay, ICU care, ventilator use etc, will communicate with participants/guardians during routine patient care. The final deidentified data analysis results may be published in literatures.
9. Identifiers might be removed and the de-identified information may be used for future research without additional informed consent from the subject.

E. <u>Chart Review</u>

1. List the data that will be used from the chart review. Alternatively, attach a separate data collection tool.
   The chart review is prospective in nature. We will collect individuals' basic characteristic and clinical variables, including date of birth, race/ethnicity, gender, insurance type, residential zip code, smoking history, height, weight, history of COVID vaccination, previous history of COVID, dexamethasone adverse events such as hyperglycemia, infections etc, respiratory support including nasal cannula oxygen, high flow oxygen, Bipap, mechanic ventilation, length of ICU stay, length of hospital stay, discharge disposition, health status 28 days after first dose of study dexamethasone eg. supplemental oxygen, tracheostomy, chronic mechanic support, daily activity limitation. We will also examine comorbidities including obesity, diabetes,

Human Research Participant Protection
The University of Oklahoma
IRB APPROVED
AAHRPP
IRB NUMBER: 12927
IRB APPROVAL DATE: 01/15/2021

hypertension, hyperlipidemia, acute kidney injury, chronic kidney disease, heart failure, arrhythmia, coronary artery disease, psychological disease etc.

2. Describe the databases that will be utilized, including specific sites from which you will collect data.

   We will collect data from Meditech database in OUMC.

3. Describe the process for obtaining and recording the records and at what point (if at all) the data is de-identified.
   The investigators conduct a review of records from their practice and collects medical records based on inclusion/exclusion criteria. Records will be de-identified during data analysis.

4. Explain how access to the data will be controlled and whether the access is logged.
   The electronic images of signed consent forms will be stored in a secured server in OUHSC campus. This secured server will be created with OUHSC IT service help. Only study team members have the access to the secured server. The data abstracted from Meditech will be store in REDCap. Only research team members have the access to REDCap.

5. What happens to the data at the end of the study?
   The electronic data will be stored in the secured server and REDCap after the project closure for future research reference.

F. Biospecimens

No biospecimens will be collected.

G. Banking/Repository/Database

Our Pulmonary Section of Department of Medicine plan to have long term research on respiratory failure due to COVID, including retrospective and prospective research. The data in this study will be important reference for future research. The electronic data will be stored in the same secured server in OUHSC. Only the study team members have the access to the data. PI will log into the server once per month to ensure the database function after the study is closed. PI will report to OUHSC IT for any concern regarding the database security. The data in REDCap will follow the regulation of OUHSC REDCap policy.

H. Inclusion / Exclusion Criteria

1. List the criteria that will define who will be **included** in the study

   Age ≥ 18 years old

Human Research Participant Protection The University of Oklahoma IRB APPROVED AAHRPP
IRB NUMBER: 12927
IRB APPROVAL DATE: 01/15/2021

PCR confirmed COVID-19 infection

Positive pressure ventilation (non-invasive or invasive) or high flow nasal cannula (HFNC) or need supplemental oxygen with oxygen mask or nasal cannula

2. List the criteria that will define who will be **excluded** in the study.
   Underlying disease requiring chronic corticosteroids
   Severe adverse events before admission, i.e. cardiac arrest;
   Contraindication for corticosteroids;
   Death is deemed to be imminent and inevitable during the next 24 hours
   Recruited in other clinical intervention trial
   Pregnancy
   Patient on judicial protection

3. Provide early termination criteria.

   The study will be terminated early if COVID pandemic ends or there is no new COVID admission to OUMC or the interim data analysis (6 weeks after study start date) shows worse primary outcome in intervention group.

I. Gender/Minority/Pediatric Inclusion for Research

Male and female adults will be included in the study. This study will be conducted in OUMC adult service. We will exclude participants age < 18 years old.

J. Recruitment and Enrollment

1. Describe the plans for recruitment.
   a. Methods to identify and recruit potential participants
      The participants will be identified when the ICU service or the hospitalist service providers are contacted for admission. The providers will review all the clinical data and decide if the potential participants meet the inclusion criteria and contact the investigators.

   b. If this is a multicenter study where subjects will be recruited by methods not under control of the local site (call centers, national advertisements), indicate this here.
      This is not a multicenter study.

2. Describe the consent procedures to be followed
   If the potential participant is able to consent him/herself (or at least assent), the potential participant will read and sign the consent form himself/herself. The paper consent form does not leave the patient room. Instead, the signature page is pressed up against the glass and the investigator takes a picture of the signature pages with his/her cell phone.
   If the potential participant is not able to consent/assent and consent is obtained from the power of attorney who is not physically present, consent is

Human Research Participant Protection The University of Oklahoma IRB APPROVED AAHRPP
IRB NUMBER: 12927
IRB APPROVAL DATE: 01/15/2021

obtained over the phone by the investigator with a witness listening. Informed Consent can be obtained verbally over the phone with a legally authorized representative and a witness, and the signed consent can be returned by fax, email or mail. The participant can be randomized and put in the study prior to return of the signed consent, however, if the signed consent is not returned, the participant will be removed from the study.

3. Describe the location where consent is most likely to take place.
   The consent will be obtained while an inpatient (ICU, ER, and medial floor)

4. Describe provisions for recruiting non-English speaking participants.
   The information on the informed consent form will be explained to patients or power of attorney and all questions will be answered using the hospital legal interpreter service before the consent form is signed.

5. Describe measures to decrease participant coercion
   The investigator allows the potential participant adequate time to review the consent, the investigator will answer all the questions the potential participant or power of attorney have before the consent form is signed.

K. <u>Risks and Benefits</u>

The risks for participants who are on dexamethasone 20mg daily group may have higher risk of dexamethasone adverse effects such as infection, hyperglycemia etc.(7) The high dose dexamethasone has been used widely in clinical practice for many years and severe adverse effects are rare. The providers will monitor the adverse effects closely and start appropriate treatment such as antibiotics and insulin. If our study shows dexamethasone 20mg daily therapy group has better health outcome, participants in this group will benefit from this therapy. On the other hand, the participants in dexamethasone 6mg daily group may not have the same good outcome as the dexamethasone 20mg daily group. Using dexamethasone 6mg daily may also cause hyper glycemia and infection etc requiring relevant treatment.

The dexamethasone 20mg*5 days, then 10mg *5 days (10 days course) costs $11.85 (vials) or $41.43 (tablets). The dexamethasone 6mg daily*10 days costs $59.1 (vials) or $131.9 (tablets). The cost of high dose therapy is lower than the low dose therapy. There should not be extra financial burden to the participants.

Although one clinical trial found dexamethasone 6mg daily for 10 days had mortality benefit compared to usual care without dexamethasone therapy,(4) the difference of health outcome between high dose and low dose therapy is not confirmed. The mental risk to participant is minimal. The investigators will explain the study in details and answer all the questions to release the stress before participants/power of attorney decide to sign the consent.

Human Research Participant Protection The University of Oklahoma IRB APPROVED AAHRPP
IRB NUMBER: 12927
IRB APPROVAL DATE: 01/15/2021

The information which can be used to identify the participant including name, date of birth will be used only to locate the data in the database. The data will be de-identified during the data analysis process. The data will be stored in a secured server in OUHSC. Only the study team members have the access to the data. Patients' personal information will not be released to public. There may not have direct benefit to the participants if our study does not reveal health outcome difference between the study groups, but the study results will help us to improve the COVID healthcare in general population.

L. Multiple Sites

This study will be conducted in OUMC only.

M. Statistical Methods

Dr. Huimin Wu, the PI and Dr. Salim Daouk, the co-investigator were trained in biostatistics and can guide and conduct the study design, sample size planning and statistical method.

A sustained clinical improvement is defined as an improvement of > 2 points using ordinal scale (table 1). There is no direct ordinal scale data available on dexamethasone 6mg to treat COVID-19. According to the RECOVERY trial, (4) 67.2% of patients in dexamethasone group were discharged from hospital within 28 days, which means at least 67.2% of the patients have clinical improvement using ordinal scale. We estimate a 2-sided α level of 0.05 and power of 80% to detect a difference of improvement of 15% or 10% between groups, the sample size is calculated in table 2. Consider the patient census in OUMC, if we choose the 15% difference of improvement, we estimate **300 patients** need to be enrolled.

Table 1. World Health Organization Ordinal Scale for Clinical Improvement (OSCI) scale for COVID-19

Descriptor	Score
Not hospitalized, no limitations on activities	1
Not hospitalized, limitation on activities	2
Hospitalized, not requiring supplemental oxygen	3
Hospitalized, requiring supplemental oxygen by mask or nasal prongs	4
Hospitalized, on non-invasive ventilation or high flow oxygen devices	5
Hospitalized, on invasive mechanical ventilation	6
Hospitalized, on invasive mechanical ventilation + additional organ support (pressors, RRT, ECMO)	7
Death	8

Table 2. Sample size calculation

2-sided		Dexamethasone 6mg	Dexamethasone 20mg	Sample

Human Research Participant Protection The University of Oklahoma IRB APPROVED AAHRPP
IRB NUMBER: 12927
IRB APPROVAL DATE: 01/15/2021

α level		improvement%	improvement%	size
of 0.05, power of 80%	15% difference of improvement	**60**	**75**	**330**
		**70**	**85**	**268**
		80	95	176
	10% difference of improvement	60	70	752
		70	80	626
		80	90	438

We will conduct descriptive statistics to characterize the study population at baseline, the health outcomes and adverse events. Continuous variables will be expressed as mean ± SD, while discrete variables will be shown as percentages. A two-tailed p value <0.05 will be considered statistically significant. Between-group comparisons will be made using the independent t-test (for 2 groups) or the ANOVA (for >2 groups). Chi-squared tests will be used to evaluate relationship of two categorical variables. Logistic regression analysis will be performed to determine the association between health outcomes (ordinal scale, length of hospital stay, ICU days, death) and risk factors, adjusted for age and PaO2:FiO2 ratio.

N. Data and Safety Monitoring Plan

Due to the urgent need for effective treatment, an internal data and safety monitoring board will be appointed for monitoring the trial progress. The board includes all project investigators. The board members will meet once per month, usually by teleconference, to review data from this trial. The board is also responsible for review of related issues, such as center performance standards. The board may request more frequent meetings if necessary to fulfill its charge. It may also request additional safety reports on a more frequent basis. The data board will review includes participants' health outcome data such as ventilator support, hospital or ICU length of stay, death, dexamethasone adverse events, such as uncontrolled hyperglycemia requiring insulin infusion, severe infection etc. After each meeting, the board will make formal recommendations regarding trial continuation and clinical performance. A special responsibility of the board is to review serious adverse events (SAEs), as defined by deaths, life threatening conditions, or events requiring permanent discontinuation of the treatment. SAEs are to be documented on case report forms and reported to the primary investigator within 24 hours of notice, with follow up reporting until the event has terminated. The SAEs will also be submitted to OUHSC IRB in a timely fashion for review. According to other clinical trial using the high dose dexamethasone, the risk of insulin use, new diagnosis of infection and SAEs were similar between intervention and placebo. (5) However, if the high dose dexamethasone group has statistically significant worse health outcome compared to the control group, it might trigger an immediate suspension of the study.

At the end of each board meeting, the board will vote whether to continue the study as planned or whether to recommend changes to the study. The board may

IRB APPROVED
IRB NUMBER: 12927
IRB APPROVAL DATE: 01/15/2021

recommend that the study be stopped early if there is evidence that the risk-benefit ratio does not warrant continuation of the trial.

O. Data Sharing

Data will not be shared with outside entity.

P. Confidentiality

The signed consent and HIPAA Forms will be kept in patient room if patients signed the forms or will be placed in the hospital confidential file box to be shredded after the document images are taken. Only electronic data will be collected. The data (identifiable and de-identified) will be stored in a secured server and REDCap in OUHSC. Only the study team members have the access to the server and review the data. Patients' personal information will not be released to public. The de-identified data for final analysis will be stored in this secured server for future research after this study is closed. The identifiable data will be stored for two years after the study is completed and will be deleted permanently by OUHSC IT department.

Q. Literature Cited

1. WHO. Global research on coronavirus disease (COVID-19). 2020. Available from: https://www.who.int/emergencies/diseases/novel-coronavirus-2019/global-research-on-novel-coronavirus-2019-ncov. Accessed Jan 1, 2021
2. WHO. Coronavirus disease (COVID-19) technical guidance: The Unity Studies: Early Investigation Protocols. 2020. Available from: https://www.who.int/emergencies/diseases/novel-coronavirus-2019/technical-guidance/early-investigations. Accessed Jan 1, 2021
3. Johns Hopkins Coronavirus Resource Center. Available from: https://coronavirus.jhu.edu. Accessed Jan 1, 2021
4. Horby P, Lim WS, Emberson JR, Mafham M, Bell JL, Linsell L, Staplin N, Brightling C, Ustianowski A, Elmahi E, Prudon B, Green C, Felton T, Chadwick D, Rege K, Fegan C, Chappell LC, Faust SN, Jaki T, Jeffery K, Montgomery A, Rowan K, Juszczak E, Baillie JK, Haynes R, Landray MJ. Dexamethasone in Hospitalized Patients with Covid-19 - Preliminary Report. N Engl J Med. 2020. Epub 2020/07/18. doi: 10.1056/NEJMoa2021436. PubMed PMID: 32678530; PMCID: PMC7383595.
5. Tomazini BM, Maia IS, Cavalcanti AB, Berwanger O, Rosa RG, Veiga VC, Avezum A, Lopes RD, Bueno FR, Silva M, Baldassare FP, Costa ELV, Moura RAB, Honorato MO, Costa AN, Damiani LP, Lisboa T, Kawano-Dourado L, Zampieri FG, Olivato GB, Righy C, Amendola CP, Roepke RML, Freitas DHM, Forte DN, Freitas FGR, Fernandes CCF, Melro LMG, Junior GFS, Morais DC, Zung S, Machado FR, Azevedo LCP. Effect of Dexamethasone on Days Alive and Ventilator-Free in Patients With Moderate or Severe Acute Respiratory Distress Syndrome and COVID-19: The CoDEX Randomized Clinical Trial. Jama. 2020;324(13):1307-16. Epub 2020/09/03. doi: 10.1001/jama.2020.17021. PubMed PMID: 32876695; PMCID: PMC7489411

IRB NUMBER: 12927
IRB APPROVAL DATE: 01/15/2021

6. Force OC-TT. OUHSC interim guidance for mangement of COVID-19 in adults pharmacotherapy information. Version 8.
7. Dexamethasone sodium phosphate injection label [updated May 2014Jan 1, 2021]. Available from: https://www.accessdata.fda.gov/drugsatfda_docs/label/2014/040572s002lbl.pdf. Accessed Jan 1, 2021

Human Research Participant Protection
The University of Oklahoma
IRB APPROVED
AAHRPP
IRB NUMBER: 12927
IRB APPROVAL DATE: 01/15/2021
